# Prophylactic Therapy of Silymarin (Milk Thistle) on Antituberculosis Drug-Induced Liver Injury: A Meta-Analysis of Randomized Controlled Trials

**DOI:** 10.1155/2019/3192351

**Published:** 2019-01-10

**Authors:** Lina Tao, Xiaoyu Qu, Yue Zhang, Yanqing Song, Si-xi Zhang

**Affiliations:** Department of Pharmacy, The First Hospital of Jilin University, Changchun 130021, China

## Abstract

**Background:**

Prophylactic therapy with silymarin to prevent the development of antituberculosis drug-induced liver injury (anti-TB DILI) has been under debate. We aimed to evaluate the effect of silymarin in the prevention of anti-TB DILI.

**Methods:**

We searched MEDLINE, PubMed, Embase, and Cochrane Central Register of Controlled Trials (CENTRAL) up to 30th November 2018. Randomized controlled trials (RCTs) that compared silymarin and placebo to prevent anti-TB DILI were included. All statistical analyses were conducted using STATA 12.0 software. Standardized mean difference (SMD) and risk ratio (RR) with 95% confidence intervals (CIs) were used to evaluate the effect of silymarin. The quality of included studies was assessed according to Cochrane handbook. Funnel plots and Egger's tests were carried out to evaluate publication bias. Sensitivity analysis was conducted to assess the influence of each study.

**Results:**

A total of 1198 patients from five RCTs (585 with silymarin and 613 with placebo groups) were included. Overall, silymarin significantly reduced the occurrence of anti-TB DILI at week 4 [RR: 0.33, 95% CI (0.15, 0.75)]. In addition, silymarin exerted protective effect on liver function in patients undergoing anti-TB drugs [SMD = − 0.15, 95% CI (−0.24, −0.07), P < 0.001 (ALT); SMD =−0.14, 95% CI (−0.23, −0.06), P = 0.001(AST); SMD =−0.12, 95% CI (−0.20, −0.03), P = 0.008 (ALP)]. Silymarin led to similar AEs in placebo groups [OR: 1.09, 95% CI (0.86, 1.39), P = 0.47].

**Conclusion:**

Prophylactic therapy of silymarin is contributed to a noticeably reduced risk of development of anti-TB DILI four weeks after the initiation. In addition, silymarin significantly improved the liver function in patients who are receiving anti-TB drugs.

## 1. Introduction

Tuberculosis (TB) is a major worldwide health threat and is one of the top 10 causes of death. In 2016, the World Health Organization (WHO) estimated that there were 10.4 million incident TB cases and 1.7 million deaths [1]. The standard combined treatment regimen of anti-TB drugs consists of isoniazid (INH), rifampicin (RIF), pyrazinamide (PZA), and ethambutol (EMB) [2].

Hepatotoxicity, one of the common adverse reactions of anti-TB drugs, varies from asymptomatic elevation of liver enzymes to fulminant hepatic failure. Anti-TB drug-induced liver injury (anti-TB DILI) leads to increased morbidity and mortality. Therefore, it may result in treatment withdrawal, drug interruption and substitution, dosage regimen adjustment, nonadherence, and drug resistance [3, 4]. The overall incidence of anti-TB DILI has been reported to be from 5% to 33%, depending on the definition of DILI and the population investigated [5].

The mechanism of anti-TB DILI remains unclear. INH causes DILI through diverse mechanisms, i.e., by the pathways of toxic metabolites, escalating oxidative stress, generation of reactive oxygen species (ROS), and lipid peroxidation [6]. RIF, an inducer of drug metabolic enzymes, triggers unconjugated hyperbilirubinemia [7]. Previous studies have reported that certain herbal drugs, phytochemicals, and food supplements can prevent and reduce the hepatotoxicity of anti-TB drugs [7].

Silymarin, a traditional herbal medicine extracted from milk thistle (*Silybum marianum* L. Gaertn) fruits, has been used as a remedy for hepatoprotection [8]. Silymarin is the collective name of flavonolignans comprised of silybin or silibinin, isosilybin, silydianin, and silychristin [8–10]. Silymarin manifests hepatoprotection by scavenging free radicals, raising the glutathione content, inhibiting lipid peroxidation, and restoring the function of enzymes, thereby generating membrane stabilization and preventing toxic metabolic liver injury [11–14].

To date, silymarin has demonstrated significant hepatoprotective effects on anti-TB DILI in animal and vitro experiments [14–16]. However, the effectiveness of silymarin is under debate [17, 18]. Some clinical studies have shown that silymarin possesses positive hepatoprotective effects [19]. In contract, other studies have observed no or limited preventive effect of silymarin [3, 20, 21]. Therefore, we performed this meta-analysis to evaluate the effect of silymarin in the prevention of anti-TB DILI. We hypothesized that use of silymarin would prevent the occurrence of anti-TB DILI in patients with TB receiving anti-TB treatment.

## 2. Methods

We followed the PRISMA (Preferred Reporting Items for Systematic Reviews and Meta-Analysis) guidelines during the preparation of this meta-analysis (Supplementary Table 1) [22]. All steps were carried out according to the Cochrane Handbook for Systematic Reviews of Interventions [23]. The present meta-analysis was not prospectively registered.

### 2.1. Search Strategy

To identify relevant randomized trials, we searched the literature through MEDLINE, PubMed, Embase, and Cochrane Central Register of Controlled Trials (CENTRAL) up to 30th November 2018 with the following search strategies: ( “silymarin” or “silibinin” or “silybum” or “silybin” or “silydianin” or “silychristin” or “milk thistle”) and (“tuberculosis” or “tubercul*∗*” or “antitubercul*∗*” or “tb”). The search was restricted to English language articles. To identify relevant unpublised studies, we searched “ISRCTN Register” and “ClinicalTrials.gov” with the same search strategies up to 30th November 2018. In addition, we searched all references in the relevant articles, abstracts, presentations or posters presented in scientific conferences, and previously published reviews for additional eligible studies.

### 2.2. Inclusion and Exclusion Criteria

Two reviewers (Qu and Zhang) independently searched for and examined the relevant studies, and discrepancies were resolved by discussion with a third author (Song). Individual studies of the RCTs on the preventive effect of silymarin on anti-TB drugs induced hepatotoxicity were included for analysis. We excluded the following articles: experimental trials researched in animals, articles focusing on pharmacokinetic or pharmacodynamic variables, and trials focusing on the in vitro activity of silymarin.

### 2.3. Data Extraction

The following data were extracted from each study: year of publication, type of trial design, number of patients, patient characteristics, treatment protocol, outcome measures, and adverse effects. Two reviewers (Qu and Zhang) independently extracted the relevant data. Disagreements were resolved by discussion with a third author (Song).

### 2.4. Quality Assessment

The methodological quality of the RCTs was evaluated according to the Cochrane Handbook for Systematic Reviews of Interventions Version 5.1.0 [23]. The assessment included the following items: sequence generation, allocation concealment, blinding of participants, personnel and outcome assessors, incomplete outcome data, selective outcome reporting, and other sources of bias. Two reviewers (Qu and Zhang) independently evaluated the quality by classifying as “high”, “low”, or “unclear” risk of bias, and disagreements were resolved by discussion with a third author (Song).

### 2.5. Outcome Measures

Outcomes were measured for the following: (1) the primary efficacy outcome was the occurrence of anti-TB DILI, which was defined by serum AST or ALT > 2 × upper normal limit (UNL); (2) the key secondary efficacy outcomes were changes in the liver enzymes, including alanine aminotransferase (ALT), aspartate aminotransferase (AST), alkaline phosphatase (ALP), and total bilirubin (TBIL); (3) the safety outcome was adverse events.

### 2.6. Statistical Analysis

The meta-analysis was performed using STATA 12.0 (Stata Corporation, College Station, TX, USA). Effect size was calculated as follows: measure at the end of follow-up – measure at baseline. Standard deviations (SDs) of the mean difference were calculated using the following formula: SD = square root [(SDbaseline)^2^  + SDfollow-up)^2^  – 2R × SDbaseline × SDfollow-up], assuming a correlation coefficient (R) = 0.5. Mean and SD values were estimated using the methods described by Wan et al. [24], provided the outcome measures were reported in median and interquartile range. We assessed heterogeneity with Q statistics generated from the *χ*^2^ test and inconsistency using the I^2^ measure. Significant heterogeneity was judged with P-values less than 0.10 or I^2^ more than 50%. We chose to adopt a Mantel-Haenszel fixed-effect model (FEM) for pooling the risk ratio (RR) or standardized mean difference (SMD) and 95% confidence interval (CI) for outcomes when heterogeneity was not significant. We chose a DerSimonian and Laird random-effects model (REM) when heterogeneity was obvious. Subgroup analyses were conducted according to inconsistent follow-up period and different study design. Sensitivity analysis was performed to test the influence of a single study on the overall effect size by the leave-one-out method. Possible publication bias in the meta-analysis was explored using Egger's test.

## 3. Results

### 3.1. Study Selection Outcomes

**Figure 1:**
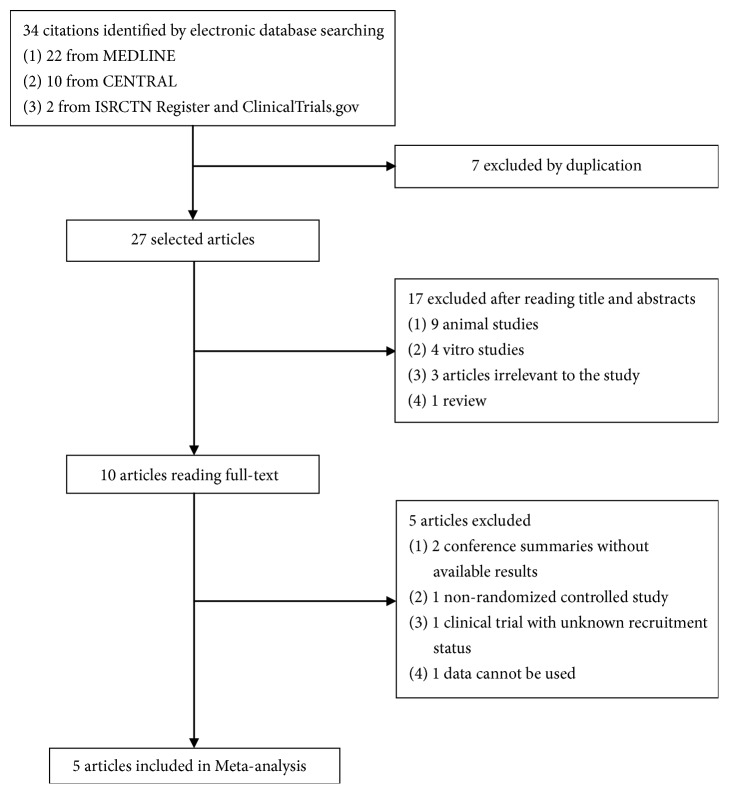
Flow diagram of literature screening and selection process.

The trial flow chart in Figure 1 shows the details of the study selection process. A total of 34 studies were identified, 17 of which were excluded based on title and abstracts, and 10 full-texts were retrieved. However, one [3] was not an RCT, seven were duplicate publications of the same study, nine [15, 25–32] were animal studies, four [14, 16, 33, 34] were in vitro research studies, three [35–37] were irrelevant to this study, one [38] was a conference summary without available results, one [39] was a clinical trial with unknown recruitment status, one [40] study's data could not be used, and one [41] was a review. Therefore, finally five RCTs [19–21, 42, 43] were included in the meta-analysis.

### 3.2. Study Characteristics and Quality Assessment

A total of 585 and 613 patients were randomly treated with silymarin and placebo, respectively.  Table 1 shows the main characteristics of the studies included in the meta-analysis.

All of the studies included in this meta-analysis were described as randomized. In two studies [20, 42], the method of randomization was not clearly described, but randomization was appropriate in other studies [19, 21, 43], which were described as computerized-based. The studies by Gu et al. in 2015 [42] and Zhang et al. in 2016 [21] were open control studies with a high risk of detection and performance bias, while the other three studies [19, 20, 43] used adequate methods to blind the intervention. All of the included studies had a low risk of incomplete outcome data. Selective reporting was low risk in the included studies because the main outcomes stated in the protocol were all reported in the final manuscript. Any other potential biases were unclear in the included studies (Table 2).

### 3.3. The Occurrence of Anti-TB DILI

**Figure 2:**
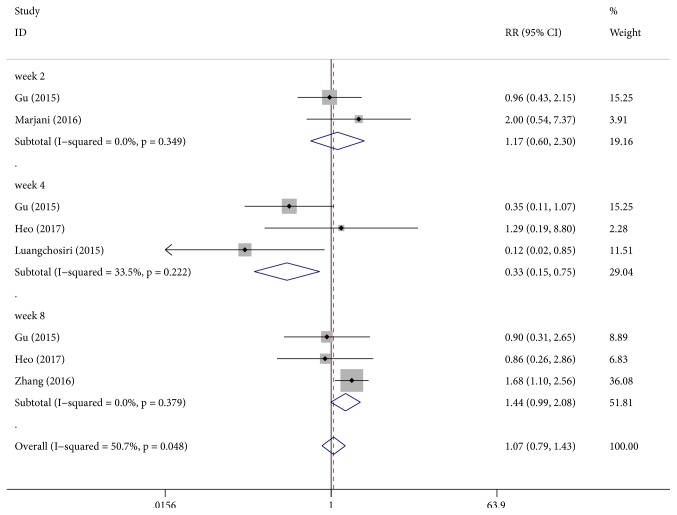
Effect of silymarin on the occurrence of anti-TB DILI with regard to time of follow-up.

All the included studies contributed to this analysis and participants were divided into three subgroups with different follow-up periods. Silymarin administration was associated with a significant reduction in the occurrence of anti-TB DILI [RR: 0.33, 95% CI (0.15, 0.75), P = 0.008] with low heterogeneity (P = 0.22, I^2^  = 33%) at week 4. No significant differences were obtained between the two groups at week 2 (P = 0.64, I^2^  = 0% ) and week 8 (P = 0.06, I^2^  = 0%) (Figure 2).

### 3.4. Changes in Liver Enzymes (ALT, AST, ALP, and TBIL)

**Figure 3:**
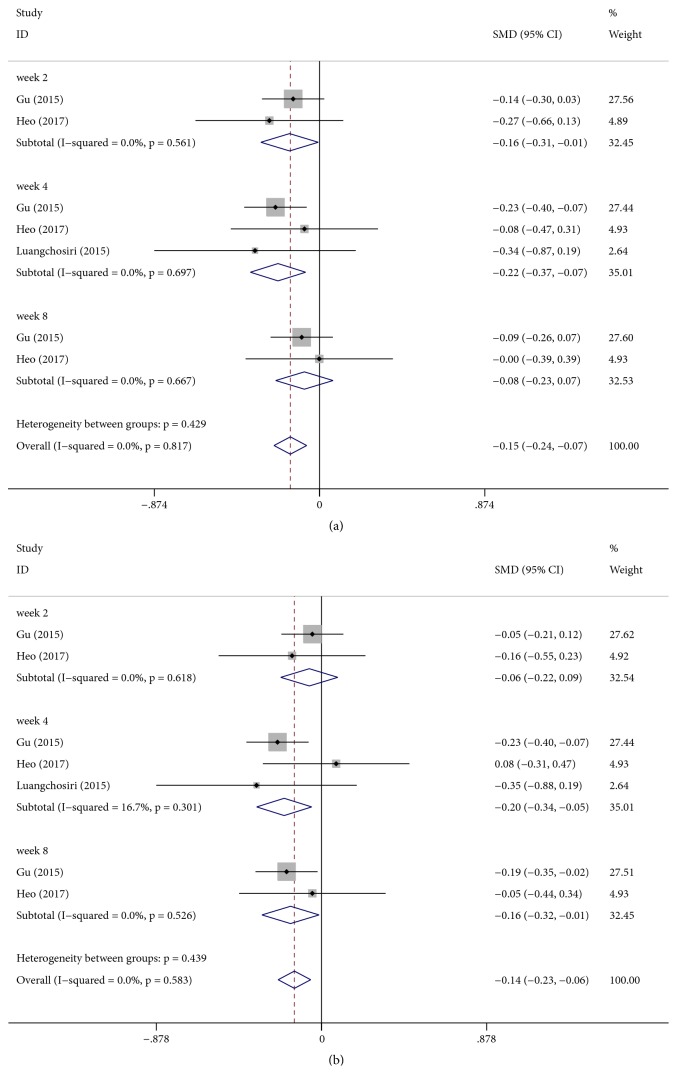
Effect of silymarin on changes in the liver function tests. (a) Alanine aminotransferase (ALT). (b) Aspartate aminotransferase (AST).

Three studies [19, 42, 43] reported the effect of silymarin on liver function enzymes. Significant differences were found with respect to change in ALT between silymarin and placebo groups [SMD: − 0.15, 95% CI (−0.24, −0.07), P < 0.001] (Figure 3(a)). In view of different follow-up durations, a subgroup analysis was performed which showed that there was no significant difference in the change in ALT level after 8 weeks of treatment [SMD: − 0.08, 95% CI (−0.23, 0.07), P = 0.30]; however, silymarin significantly decreased ALT levels compared with placebo groups after 2 weeks of treatment [SMD: − 0.16, 95% CI (−0.31, −0.01), P = 0.04] and after 4 weeks of treatment [SMD: − 0.22, 95% CI (−0.37, −0.07), P = 0.003].

There was a significant difference between silymarin and placebo groups in terms of AST change [SMD: −0.14, 95% CI (−0.23, −0.06), P = 0.001] (Figure 3(b)). Because of different follow-up durations, we performed a subgroup which showed that there was no significant difference in the change in AST level after 2 weeks of treatment [SMD: − 0.06, 95% CI (−0.22, 0.09), P = 0.40]; however, silymarin noticeably decreased AST levels compared with placebo groups after 4 weeks [SMD: − 0.20, 95% CI (−0.34, −0.05), P = 0.008] and after 8 weeks of treatment [SMD: − 0.16, 95% CI (−0.32, −0.01), P = 0.034].

**Figure 4:**
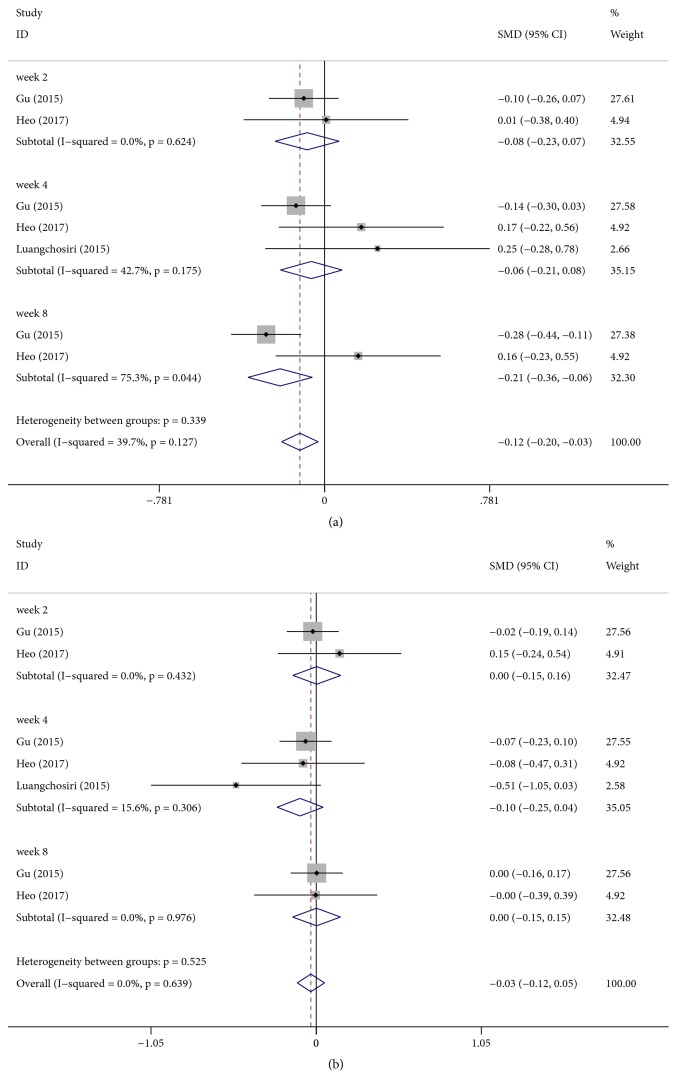
Effect of silymarin on changes in the liver function tests. (a) Alkaline phosphatase (ALP). (b) Total bilirubin (TBIL).

There was a significant difference between silymarin and placebo groups in terms of ALP change [SMD: −0.12, 95% CI (−0.20, −0.03), P = 0.008] (Figure 4(a)). Because of different follow-up durations, we performed a subgroup analysis which showed that there were no significant differences in the change in ALP level after 2 weeks of treatment [SMD: − 0.08, 95% CI (−0.23, 0.07), P = 0.288] and after 4 weeks of treatment [SMD: − 0.06, 95% CI (−0.21, −0.08), P = 0.40]; however, silymarin noticeably decreased ALP level compared with placebo group after 8 weeks of treatment [SMD: − 0.21, 95% CI (−0.36, −0.06), P = 0.007].

There was no significant difference between silymarin and placebo groups in terms of TBIL change [SMD: −0.03, 95% CI (−0.12, 0.05), P = 0.441] (Figure 4(b)).

### 3.5. Adverse Event Analysis

**Figure 5:**
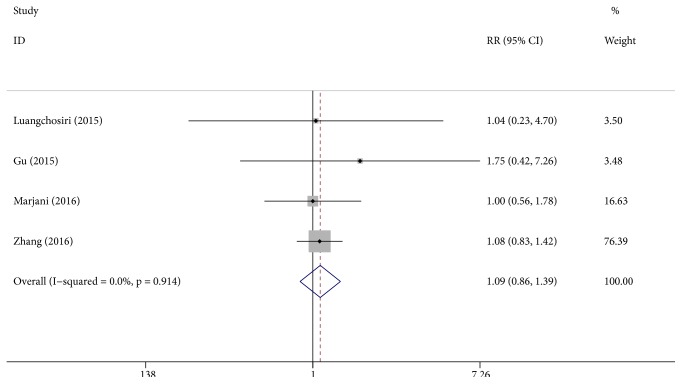
Effect of silymarin on adverse events in patients undergoing anti-TB treatment.

Adverse events were reported in four studies [19–21, 42]. Nausea/vomiting was the most frequently reported adverse event [19–21, 42]. Abdominal distension/pain and anorexia were also commonly reported [20, 42]. Rash/exanthema was observed in two studies [20, 21]. Dizziness and pruritus were also observed in two studies [19, 20]. There was no significant difference in the proportion of patients with adverse events in both groups [RR: 1.09, 95% CI (0.86, 1.39), P = 0.47] and with no heterogeneity (P = 0.91, I^2^  = 0%) (Figure 5).

### 3.6. Subgroup Analysis

Stratified analysis was also performed based on blinded or open-labelled study designs to explore potential sources of heterogeneity among studies that considered the occurrence of anti-TB DILI as an example. No heterogeneity was found, and the open-labelled study design did not alter the direction of the pooled effect (Supplementary Figure 1(a), Supplementary Figure 1(b)).

### 3.7. Sensitivity Analysis and Publication Bias

In the sensitivity analysis, we successively eliminated studies one-by-one to recalculate RR, which showed one obvious fluctuation (Supplementary Figure 2(a)). No significant publication bias was detected with Egger's test (P=0.093). The funnel plot showed symmetry on visual inspection (Supplementary Figure 2(b)).

## 4. Discussion

Anti-TB DILI is an important and pivotal adverse effect that can occur during the initial two months when combination therapy with three to four anti-TB drugs is required [19]. Any effective measure that can prevent anti-TB DILI is of significance. Therefore, we conducted this meta-analysis to evaluate the effect of silymarin on prevention of anti-TB DILI.

In this meta-analysis, the efficacy of silymarin was assessed by comparing the incidence of anti-TB DILI and changes in the liver enzymes. Fortunately, silymarin reduced the occurrence of anti-TB DILI at week 4 [OR: 0.33, 95% CI (0.15, 0.75), P = 0.008]. Additionally, it reduced ALT levels at weeks 2 and 4, and AST levels at weeks 4 and 8. Moreover, reduction in ALP level at week 8 was significant. However, the effect of silymarin on TBIL level was similar to that of placebo.

A previous study indicated the median interval from treatment initiation of anti-TB drugs to development of clinical symptoms to be 16 weeks (range 6 weeks to 6 months) [44]. According to a recent Korean cohort study focusing on the time of onset of anti-TB DILI that included 1031 TB patients, the majority (67.6%) hepatotoxic events appeared within the first 30 days of anti-TB treatment [45]. This meta-analysis suggested that silymarin showed efficacy in the prevention of anti-TB DILI. Interestingly, silymarin administration significantly reduced the incidence of anti-TB DILI at week 4; however there was no beneficial effect on the incidence at week 8. This showed that silymarin shows hepatoprotective effect during short-term treatment of anti-TB drugs. Previous research studies reported that anti-TB drugs result in oxidative stress, lipid peroxidation, and exhaustion of glutathione reserves [7, 44]. Recent studies supported that INH-induced hepatotoxicity has also been attributed to a reactive metabolite and an immune mediated reaction [6, 46]. These intricate mechanisms responsible for anti-TB DILI may be partly neutralized by the mechanism of hepatoprotection of silymarin. Furthermore, there may be a compensatory or adaptive response against anti-TB DILI, representing immune tolerance that prevents further liver injury, which could interpret delayed onset of DILI [47]. In general, silymarin significantly reduced the incidence of anti-TB DILI at week 4.

Silymarin administration significantly lowers serum AST and ALP levels. Despite silymarin presenting beneficial effects on lowering the serum AST and ALP levels at week 8, it could not reduce the incidence of anti-TB DILI at week 8. This proved that silymarin has no promising hepatoprotective effect in the long-term duration treatment with anti-TB drugs.

Considering the safety of silymarin, only minor adverse effects such as nausea/vomiting, abdominal distension/pain, anorexia, rash/exanthema, and dizziness were reported. All of these reported adverse events were mild and tolerable. Moreover, it showed no significant difference with placebo groups.

There were some limitations to our study. First, the sample size was relatively small with only 585 patients in the silymarin group and 613 in the placebo group. Second, we included two open-labelled studies [21, 42], which may give rise to information bias for the lack of blinding. However, the outcomes were not affected by the absence of blinding. Third, one study [21] chose vitamin C, a potential hepatoprotector [48], as the control instead of a placebo, which may lower the preventive effect of silymarin in anti-TB DILI. In addition, silymarin is a multi-ingredient product, and its effect may vary due to differences in cultivars, active ingredients, and methods of improving low bioavailability [49, 50]. Therefore, further large-scale, well-designed clinical trials are required to confirm and validate the preventive effect of silymarin on anti-TB DILI.

## 5. Conclusions

Drug-induced liver injury associated with antituberculosis drugs is a common adverse event. It is essential to prevent the occurrence of anti-TB DILI, because anti-TB DILI may affect treatment compliance and therapeutic effectiveness in patients with tuberculosis. This meta-analysis suggested that silymarin showed moderate efficacy in the prevention of anti-TB DILI, as it significantly reduced risk of development of anti-TB DILI at week 4, and decreased serum ALT levels at weeks 2 and 4, serum AST levels at weeks 4 and 8, and ALP level at week 8. In addition, silymarin was well-tolerated. The intricate mechanisms of anti-TB DILI have been poorly understood. Moreover, further studies on the effect of silymarin on the prevention of anti-TB DILI are needed with respect to consensus definition of anti-TB DILI and homogeneous follow-up period.

## Figures and Tables

**Table 1:** Baseline characteristics of the trials included in the meta-analysis.

study	Luangchosiri et al. 2015 [19]	Marjani et al. 2016 [20]	Zhang et al. 2016 [21]	Gu et al. 2015 [42]	Heo et al. 2017 [43]
design	Randomized double-blind controlled trial	randomized double blind	randomized controlled open labelled	open-label randomized multi-center	Prospective Randomized double-blind placebo-controlled
patients randomized	58	72	379	568	121
age	56.00	50.10	< 41 years, 180 *⩾*41 years, 190	37.42 ± 14.28	57.73 ± 13.94
male	22(40.00)	37 (52.86)	274(74.05)	374(65.85)	68(66.02)
diagosis	pulmonary TB	diagnosed of TB	diagnosed of TB	diagnosed of TB and having primary pulmonary TB	diagnosed of TB
anti-TB regimen	standard regimen consisting isoniazid (5 mg/kg), rifampin (10 mg/kg), pyrazinamide (25 mg/kg) and ethambutol (15 mg/kg)	standard regimen consisting isoniazid (5 mg/kg), rifampin (10 mg/kg), pyrazinamide (20 mg/kg) and ethambutol (15 mg/kg)	the standard anti-tuberculosis therapy including isoniazid (H), rifampicin (R), pyrazinamide (Z), and thambutol (E)	the standard anti-TB treatment drugs including isoniazid (H), rifampicin (R), pyrazinamide (Z), and thambutol (E)	the first line standard anti-TB treatment drugs including isoniazid, rifampicin, ethambutol and pyrazinamide
experimental group	silymarin, 140 mg, tid	sylibum marianum ( equivalent to 140 mg silymarin), tid	silybum marianum, 200 mg, bid	silibinin, 70 mg, tid	silymarin, 140 mg, bid
control group	placebo, tid	placebo, tid	vitamin C, bid	none	placebo, bid
follow-up	week 2, 4	week 2	week 8	Week2, 4, 8	Week 2, 4, 8
Country	Thailand	Iran	China	China	Korea
Outcomes	①②④	①④	①②③④	①②③④	①②③

Values are presented as number (%) or mean±SD.

①: the occurrence of anti-TB treatment related DILI; ②: liver function tests (ALT, AST, ALP, and TBIL); ③: the occurrence of interruption of anti-TB treatment or taking the second-line TB drugs; ④: adverse events.

**Table 2:** Risk of bias assessment in the studies included for meta-analysis.

Study	Random sequence generation (selection bias)	Allocatrion concealment (selection bias)	Blinding of participants and personnel (performance bias)	Blinding of outcome assessment (detection bias)	Incomplete outcome data(attrition bias)	Selective reporting (reporting bias)	Other types of bias
Luangchosiri et al. 2015 [19]	L	L	L	L	L	L	U
Marjani et al. 2016 [20]	U	U	L	L	L	L	U
Zhang et al. 2016 [21]	L	H	H	H	L	L	U
Gu et al. 2015 [42]	U	H	H	H	L	L	U
Heo et al. 2017 [43]	L	L	L	L	L	L	U

Criteria defined for quality assessment are based on the Cochrane guidelines.

H: high risk of bias; L: low risk of bias; U: unclear risk of bias.

## Supplementary Materials

Supplementary Materials Supplementary Figure 1: subgroup analysis of the occurrence of anti-TB DILI based on blinded or open-labelled study designs. (a) Follow-up at week 4. (b) Follow-up at week 8. Supplementary Figure 2: (a) forest plot of sensitivity analysis in the meta-analysis; (b) funnel plot for the effect of silymarin on the occurrence of anti-TB DILI. Supplementary Table 1: PRISMA 2009 Checklist.
